# Effect of ProRoot MTA^®^ and Biodentine^®^ on osteoclastic differentiation and activity of mouse bone marrow macrophages

**DOI:** 10.1590/1678-7757-2018-0150

**Published:** 2019-01-07

**Authors:** Miri Kim, Soojung Kim, Hyunjung Ko, Minju Song

**Affiliations:** 1 Asan Medical Center, University of Ulsan, Department of Conservative Dentistry, Seoul, Korea; 2 Dankook University, College of Dentistry, Department of Conservative Dentistry, Cheonan, Korea

**Keywords:** Biodentine, ProRoot MTA, Alendronate, Osteoclastogenesis

## Abstract

**Objectives:**

This investigation aimed to assess the differentiation inhibitory effects of ProRoot MTA^®^ (PMTA) and Biodentine^®^ (BIOD) on osteoclasts originated from murine bone marrow macrophages (BMMs) and compare these effects with those of alendronate (ALD).

**Materials and Methods:**

Mouse BMMs were cultured to differentiate into osteoclasts with macrophage colony-stimulating factor and receptor activator of NF-κB (RANKL), treated with lipopolysaccharide. After application with PMTA, BIOD, or ALD, cell toxicities were examined using WST-1 assay kit, and RANKL-induced osteoclast differentiation and activities were determined by resorption pit formation assay and tartrate-resistant acid phosphate (TRAP) staining. The mRNA levels of osteoclast activity-related genes were detected with quantitative real time polymerase chain reaction. Expressions of molecular signaling pathways were assessed by western blot. All data were statistically analyzed with one-way ANOVA and Tukey's post-hoc test (p<0.05).

**Results:**

Mouse BMMs applied with PMTA, BIOD, or ALD showed highly reduced levels of TRAP-positive osteoclasts. The BIOD treated specimens suppressed mRNA expressions of cathepsin K, TRAP, and c-Fos. Nonetheless, it showed a lower effect than PMTA or ALD applications. Compared with ALD, PMTA and BIOD decreased RANKL-mediated phosphorylation of ERK1/2 and IκBα.

**Conclusions:**

PMTA and BIOD showed the inhibitory effect on osteoclast differentiation and activities similar to that of ALD through IκB phosphorylation and suppression of ERK signaling pathways.

## Introduction

Root resorption, a complication of Dentistry, may occur after dental trauma, surgical procedure, orthodontic treatment, or bleaching.^1^ A large area of damaged root surface and continual resorption process may result in the loss of teeth. As resorption is mediated by osteoclasts/odontoclasts, reducing osteoclastic activity is a key to inhibit the progression of root resorption. Several studies have reported a method to suppress the progression of root resorption. In cases of avulsion, or surgical procedure of intentional replantation, the application of fluoride, dexamethasone, or other solutions is recommended.^2^ ^,^ ^3^

Osteoclasts (OCLs) are multinucleated giant cells resulted from a hematopoietic monocyte or macrophage lineage.^4^ Odontoclasts are multinucleated giant cells involved in the resorption of hard dental structures and are similar to OCLs regarding cellular origins, characteristics, and function including the dissolution of mineralized hard tissues.^5^ ^,^ ^6^ In this respect, anti-resorptive drugs have been used for patients with osteoporosis, e.g. bisphosphonate was used to inhibit root resorption.^7^ ^,^ ^8^ Bisphosphonates are a family of popular anti-resorptive agents. A family member, alendronate (ALD), exhibited increased anti-resorptive capacity upon the addition of nitrogen into the side chain.^9^ In addition to ALD, nitrogen-containing bisphosphonates also include zolendronate, risedronate, ibandronate, and others.^10^ Local treatment with ALD has been shown to prevent root resorption by inhibiting macrophages.^11^ ^,^ ^9^

Calcium silicate-containing cements (CSCs) are widely applied in endodontic treatments, not only as a retrograde filling material, but also for pulp capping material, apexification, and re-vascularization procedures.^12^ ^,^ ^13^ Recent studies have reported the calcium silicate-containing mineral trioxide aggregate (MTA) inhibits OCL differentiation and bone resorption, and possesses good biocompatibility and bioactivities to promote healing mechanisms.^14^ ^,^ ^15^ ^,^ ^11^ Biodentine^®^ (BIOD; Septodont, Saint-Maur-des-Fossés, France), a novel biocompatible repair and dentine-like components to MTA have been used since 2011.^16^ BIOD is considered to be an alternative to MTA with comparable outcomes in various clinical applications.^17^ ^,^ ^16^

This investigation aimed to assess the differentiation effects of ProRoot MTA^®^ (PMTA) and BIOD on OCLs originated from murine bone marrow macrophages (BMMs), and compare these effects with those of ALD. As few researches have examined the anti-resorptive activity of BIOD, the results of this study may support the application of CSCs as a choice of treatment to prevent root resorption.

## Material and methods

### Agent preparation

The two CSCs used in this investigation, BIOD and PMTA (PMTA; Dentsply, Tulsa, OK, USA), and 10^-8^ mol/L ALD (Santa Cruz Biotechnology Inc., Santa Cruz, CA, USA) were prepared as described in previous studies.^18^ ^,^ ^19^ Briefly, BIOD and PMTA were mixed according to their respective manufacturer's instructions and packed into sterilized plastic molds (5 mm inner diameter, 2 mm thickness). Once set, discs were removed from the molds and sterilized with ethylene oxide gas. Two discs of each material were then placed into 4 mL of α-Minimum Essential Medium (α-MEM; HyClone, Logan, UT, USA) containing 1% penicillin-streptomycin (Gibco, Grand Island, NY, USA) in a glass vial, and incubated at 37°C for 72 h. After incubation, conditioned media were collected and passed through a 0.22 μm filter.

### Cell culture method

Six week-old male ICR mice were obtained from Koatech (Pyeongtaek, Korea). Primary mouse bone marrow cells (BMCs), obtained by flushing femurs and tibia, were cultured in α-MEM containing 10% fetal bovine serum (FBS; Gibco, Grand Island, NY, USA), and 1% penicillin and streptomycin. After culturing for 24 h, nonadherent cells were harvested and cultured with 30 ng/mL of macrophage colony-stimulating factor (M-CSF) to produce BMMs. Next, BMMs were cultured to differentiate into OCLs with 30 ng/mL of M-CSF and 30 ng/mL of receptor activator of NF-kB (RANKL; R&D Systems, Minneapolis, MN, USA) for more than 4 days. To promote differentiation, 100 ng/mL of lipopolysaccharide (LPS, *Escherichia coli* O26:B6; Sigma-Aldrich, St. Louis, MO, USA) was added after 48 h of M-CSF/RANKL treatment. BMMs without RANKL/LPS ((-)CTL) and BMMs with RANKL/LPS ((+)CTL), i.e. OCLs, were negative and positive controls, respectively. Finally, OCLs were treated with BIOD conditioned media, PMTA conditioned media, or media with 10^-8^ mol/L ALD. All experiments were performed with these OCLs.

### Cytotoxicity assay

Cell viability was evaluated using an EZ-Cytox cell viability assay kit (WST method; DoGenBio, Seoul, Korea) according to the company's instructions. OCLs at a density of 2x10^4^ cells/mL were seeded into 96-well plates containing each agent. At 48 h and 72 h after cell-seeding, 10 μL of WST solution was added to each well. Cells were then incubated for additional 3 h. Before investigating the plate, it was well shaken for 1 min for a homogeneous color distribution. Then, optical density (OD) was measured at a wavelength of 450 nm using a VersaMax micro-plate reader (Molecular Devices, Sunnyvale, CA, USA). Consequently, we could calculate the final value using the following formulas: total signal − background signal = original signal; original signal ÷ control signal = viability.

### Tartrate-resistant acid phosphatase (TRAP) staining

BMMs were seeded in 96-well plates at a density of 1x10^4^ cells *per* well and *cultured* to differentiate into OCLs. After incubation with or without each agent for 24 h, cells were washed with phosphate buffered saline (PBS) and fixed for 10 s with liquid containing citrate, acetone, and formaldehyde. Next, cells were stained using TRAP kit solution (Sigma, St. Louis, MO, USA) for 10 – 20 min. TRAP positive cells appeared dark red and were identified according to the presence of multiple nuclei (n>3). OCLs were counted in triplicate cultures using Image-J software version 1.48 (National Institutes of Health, Bethesda, MD, USA) on digital images acquired with a Nikon Eclipse (TS100, Tokyo, Japan) at 100× magnification.

### Resorption pit formation assay

BMMs were seeded in 96-well plates previously equipped with dentin slices at a density of 1x10^4^ cells *per* well, and subsequently cultured to differentiate into OCLs. After incubation with or without each agent for 72 h, cells were flushed twice with PBS and then scraped off the dentin slices. Slices were then stained with TRAP staining solution. Stained pit formation was photographed.

### RNA isolation and quantitative real-time PCR (qRT-PCR)

**Figure 1:**
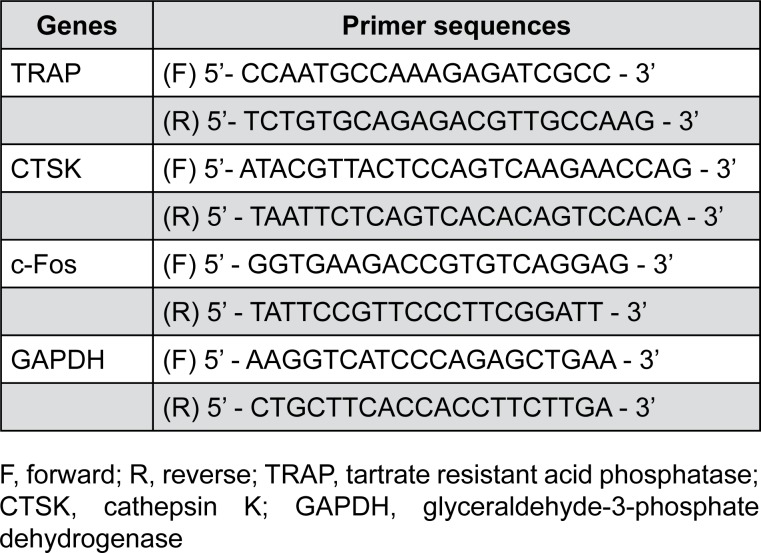
Primer sequences for qRT-PCR analysis

After a 24-h treatment with or without each agent, total RNA was extracted from cultured cells using a RNeasy Plus Mini Kit (Qiagen, Hilden, Germany) and analyzed for osteoclastic markers: TRAP, cathepsin K (CTSK), c-Fos, and GAPDH. RNA samples (1 μg) were reverse-transcribed for cDNA synthesis using a Maxime RT PreMix Kit (iNtRON Biotech., Seongnam, Korea) according to the manufacturer's instructions. cDNA synthesis was carried out at 45°C for 1 h, and then incubated at 95°C for 5 min to stop the enzyme reaction. The RT product was diluted with 5–20 μL sterile water. Figure 1 listed the sequences of primers used for PCR amplification. Data were calibrated to GAPDH expression and are shown as the mean ratio ± SD from triplicate experiments.

### Western blot

BMMs were seeded into 6-well plates at a density of 5x10^4^ cells/ml and induced to differentiate into OCLs with or without each agent. Cells were flushed twice with PBS and lysed using radioimmunoprecipitation assay buffer (Tech and Innovation, BRI-9001, Chuncheon, South Korea) with protease inhibitor cocktail tablets (Roche, Mannheim, Germany). Cells were scraped off and incubated on ice for 10 min. Cell debris was sonicated and centrifuged at 20,000 × g at 4°C for 20 min, and the supernatant was collected for western blot analysis. After electrophoresis using a 10% SDS-PAGE gel, proteins were transferred to immobilized membranes (Millipore, Darmstadt, Germany), and then blocked with 5% non-fat milk for 1 h at room temperature. Next, membranes were incubated with the following primary antibodies: anti-phospho-p44/42 MAPK (Erk1/2) (Thr202/Tyr204), anti-phospho-IκBα (Ser32/36) (5A5), anti-p44/42 MAPK (Erk1/2) (137F5), and anti-IκBα (L35A5) (Cell Signaling Technology, Beverly, MA, USA). Subsequently, membranes were probed with species-specific horseradish peroxidase-conjugated secondary antibodies. Signals were examined using an ImageQuant LAS 4000 mini (GE Healthcare Life Sciences, Björkgatan, Sweden). Phosphorylation of IκB and ERK1/2 was determined by western blotting of OCLs at 15 and 30 min. Then, membranes were stripped and antibodies against non-phosphorylated IκB and ERK1/2 were used as a control.

### Statistical analysis

All data were derived from triplicates in each of the three independent experiments. Data are presented as mean±standard deviation (SD). Data were statistically analyzed using one-way analysis of variance followed by Tukey's *post-hoc* test. Statistical significance between groups was set at 5% (P<0.05).

## Results

### Cytotoxicity assay

**Figure 2:**
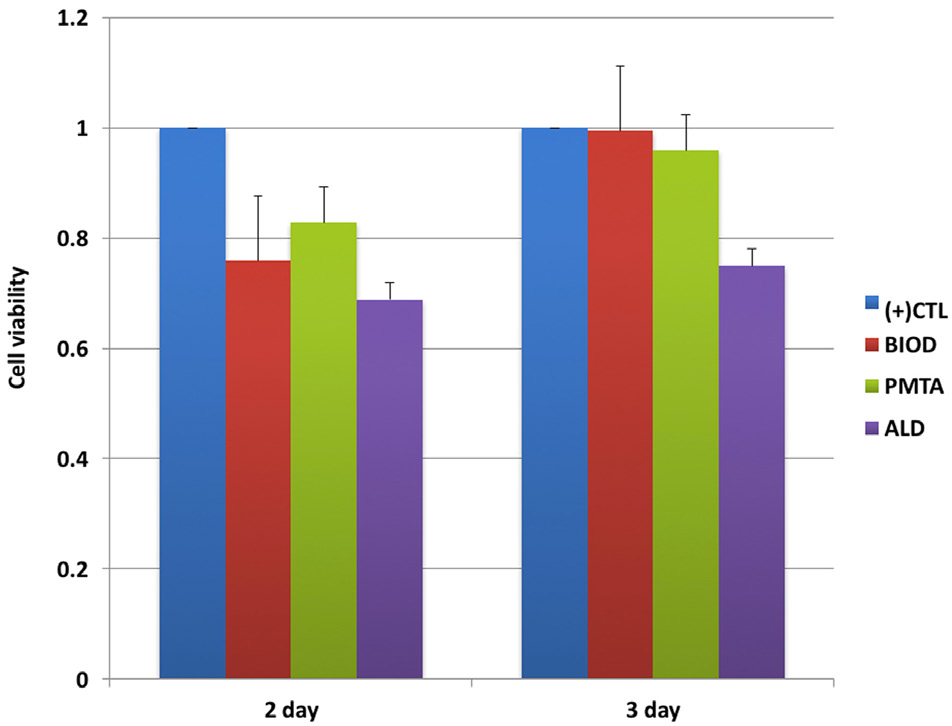
Bone marrow macrophages (BMMs) were incubated with drug-treated media for 48 h or 72 h, after which the viability of cells was assessed using a WST assay. Treatment with Biodentine (BIOD), ProRoot MTA (PMTA), or alendronate (ALD) resulted in decreased cell viability compared with (+) CTL

Viability of OCLs after exposure to agents for 48 and 72 h was assessed. WST assays showed the cytotoxicity of agents did not affect cell viability (Figure 2). Cytotoxicity results showed no significant differences in cell viability (P>0.05). However, ALD presented relatively high cytotoxicity to BMMs compared with CSCs.

### TRAP staining

**Figure 3:**
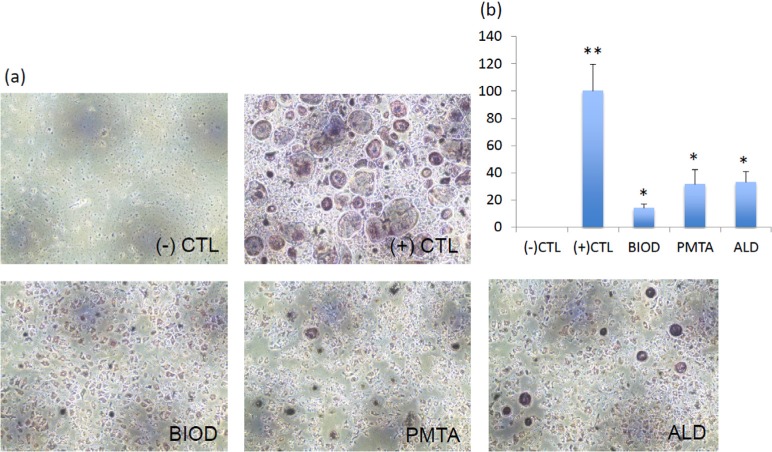
Tartrate-resistant acid phosphate (TRAP) staining of osteoclasts (OCLs) treated with or without CSCs or ALD. (a) Representative images of each group. (-) CTL, BMMs without RANKL/LPS treatment; (+) CTL, OCLs (BMMs treated with RANKL/LPS); BIOD, OCLs treated with Biodentine conditioned media; PMTA, OCLs treated with ProRoot MTA conditioned media; ALD, OCLs treated with alendronate. (b) Quantification of TRAP-positive osteoclasts. Number of TRAP-positive cells was significantly higher in (+) CTL (P<0.05), while no significant differences were observed between BIOD, PMTA, and ALD (P>0.05). Different asterisks (* and **) means that they were significantly different each other. BMMs=bone marrow macrophages; CSCs=calcium silicate-based cements; LPS=lipopolysaccharide; OCLs=osteoclasts; RANKL=receptor activator of NF-κB; TRAP=tartrate-resistant acid phosphate

Compared with negative control BMMs, RANKL/LPS-induced BMMs differentiated into mature TRAP positive multinucleated OCLs, which served as a positive control. However, numbers of TRAP positive OCLs decreased upon treatment with each agent. The number of TRAP positive cells was significantly higher in the positive control group (P<0.05), while no significant differences were observed between OCLs treated with agents (P>0.05) (Figure 3). BIOD showed the least TRAP-positive cells, although no statistical significance was observed. Thus, CSC treatment inhibited RANKL/LPS-induced differentiation of BMMs into OCLs at a level similar to ALD.

### qRT-PCR

**Figure 4:**
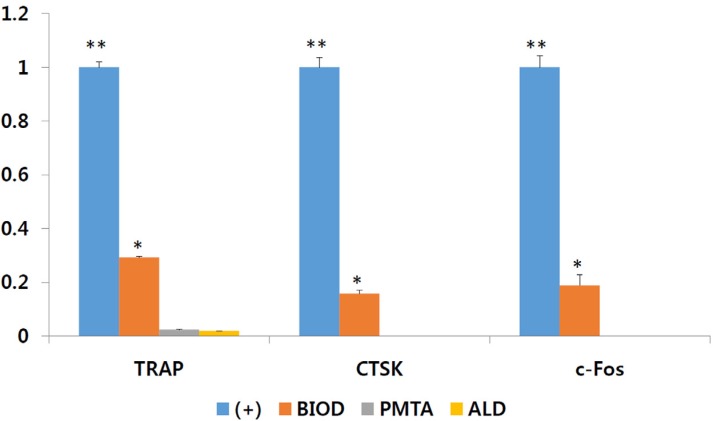
CSCs and ALD exhibited an inhibitory effect on osteoclast differentiation and function. (A) mRNA expression of osteoclastic markers TRAP, CTSK, and c-Fos were analyzed by qRT-PCR. mRNA expression of osteoclastic markers was decreased in BIOD, PMTA, and ALD compared with (+) CTL (P<0.05). Different asterisks (* and **) means that they showed significantly different each other. ALD=alendronate; BIOD=Biodentine; CSCs=calcium silicate-based cements; CTSK=cathepsin K; PMTA=ProRoot MTA; TRAP=tartrateresistant acid phosphate

To further examine changes in the differentiation of RANKL/LPS-mediated OCLs induced by CSCs, the expression of osteoclastogenesis-related genes was determined by qRT-PCR (Figure 4). mRNA expression of osteoclastic markers decreased in OCLs treated with CSCs compared with the positive control (P<0.05), demonstrating that the agents used in this study affect osteoclastic differentiation of OCLs. Compared with BIOD, PMTA showed a similar tendency to ALD regarding significantly down-regulated expression of TRAP, CTSK, and c-Fos (P<0.05).

### Resorption pit assay

**Figure 5:**
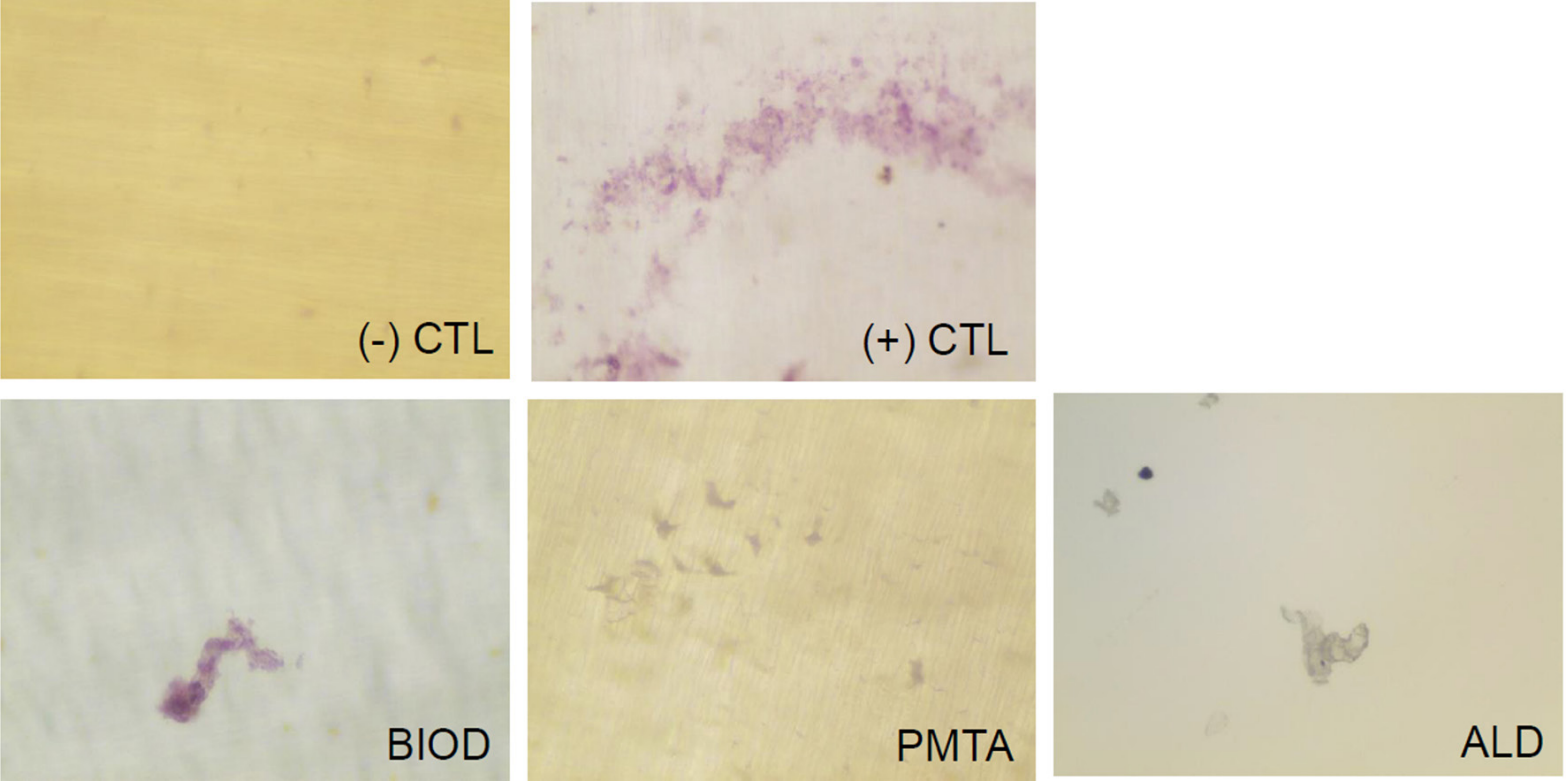
Resorptive activity of OCLs treated with or without CSCs or ALD. CSCs and ALD showed an inhibitory effect on osteoclast function. Pit formation was confirmed as something purple on the smooth disc. ALD=alendronate; BIOD=Biodentine; CSCs=calcium silicate-based cements; OCLs=osteoclasts; PMTA=ProRoot MTA

To determine whether CSC treatment affected the activity of OCLs, the resorption area was examined. After 3 days incubation on the dentin disc, mature OCLs showed stained pits with purple color (Figure 5). CSCs and ALD inhibited the function of mature OCLs, as indicated by fewer pits on the discs.

### Western blot analysis

**Figure 6:**
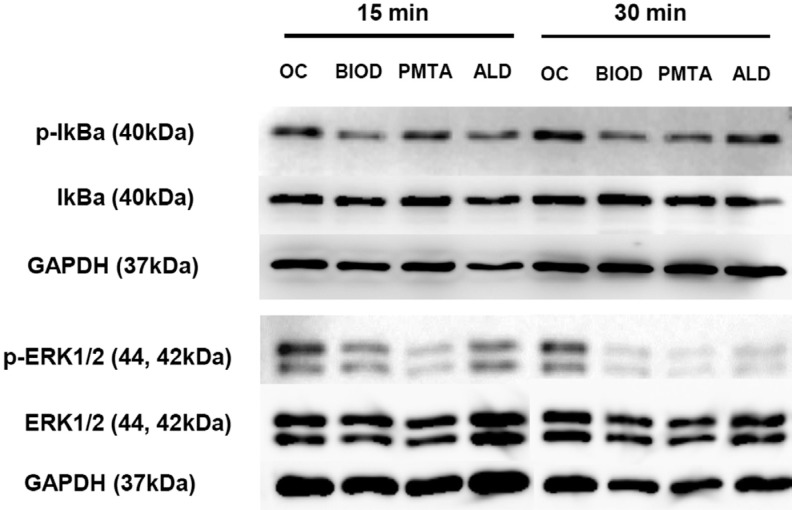
CSCs and ALD suppressed activation of the NF-kB pathway in RANKL/LPS-stimulated BMMs. ALD=alendronate; BIOD=Biodentine; BMMs=bone marrow macrophages; CSCs=calcium silicate-based cements; LPS=lipopolysaccharide; OCLs=osteoclasts; PMTA=ProRoot MTA; RANKL=receptor activator of NF-êB

To investigate the activation of NF-kB that drives RANKL-mediated osteoclastogenesis, IκBα phosphorylation was assessed by western blotting (Figure 6). CSCs and ALD treatment decreased IκBα phosphorylation in OCLs. MAPK activities were examined by assessing ERK1/2 phosphorylation 15 and 30 min after treatment. CSCs and ALD also significantly inhibited RANKL-mediated phosphorylation of ERK1/2.

## Discussion

Root resorption is mainly caused by OCLs (i.e. odontoclasts). ALD, a nitrogenous bisphosphonate, is an effective anti-resorptive drug for inhibiting OCL activity. Many studies have demonstrated the utility of ALD for preventing root resorption of replanted avulsed teeth or inhibiting inflammatory bone resorption.^7^ ^,^ ^8^ ^,^ ^20^ We also demonstrated that ALD treatment inhibited osteoclastic activity in OCLs derived from mouse BMMs, consistent with previous studies. Nevertheless, it was reported that amino-containing bisphosphonates, such as alendronic acid, possess pro-inflammatory properties that can trigger the production of pro-inflammatory cytokines and initiate acute-phase inflammation.^21^ ^,^ ^22^ Putranto, et al.^23^ (2008) reported that ALD transiently increased root pit formation because of increased odontoclast numbers.

CSCs, including the first-developed ProRoot MTA®, are materials widely applied in Dentistry and have the advantage of osteoinductivity. In addition, recent studies have reported that CSCs prevent osteoclastogenesis and bone resorption to enhance bone remodeling, suggesting that silicate may take part in the inhibitory behavior of CSCs on osteoclastogenesis.^24^ ^,^ ^14^ CSCs show varying degrees of bioactivity depending on their composition, which may affect the ion-releasing property of a material. In this regard, it was recently reported the amount of silicate dissolution could be larger in BIOD than in MTA, as more substantial uptake of calcium and silicate by adjacent bovine dentine has been shown.^25^ Lately, an *in vivo* study reported osteoinductive potential, bone bonding ability and biomineralization of newly developed CSCs, including BIOD.^26^ BIOD showed osteoinductive properties presenting replacement by novel bone. Nonetheless, the inhibitory effect of this newly developed bioceramic on osteoclastic activity was still obscure. Therefore, we aimed to compare the effect of BIOD on osteoclastic activity with PMTA and ALD using murine BMMs, and hypothesized that CSCs such as BIOD could be considered an alternative to ALD for inhibition of root resorption.

RANKL, a component of the tumor necrosis factor superfamily expressed by the stromal environment, binds to its functional receptor, RANK, on OCLs to induce osteoclastogenesis. Indeed, RANKL/RANK signaling plays a crucial role in OCL differentiation, activation, and survival.^27^ ^,^ ^28^ LPS, a composition of Gram-negative bacterial cell walls, is a famous pathogen of inflammatory bone resorption. LPS reportedly stimulates the survival and fusion of OCL progenitors independent of RANKL,^29^ and reportedly induces OCL formation.^30^ Most root resorptions involve in the inflammatory environment of pulp and/or periapical tissue. Therefore, we treated BMMs sequentially with M-CSF, RANKL, and LPS to form OCLs in this study.

In accordance with a previous study,^19^ neither CSCs nor cements containing antiosteoporotic drugs were cytotoxic to BMMs and OCLs, as evaluated by WST assay. Given that local treatment of ALD can compromise the revascularization of the alveolus around the replanted teeth after avulsion,^9^ non-toxic concentrations (≤10^−8^ mol L^-1^) were applied to determine the effects of ALD on osteoclastogenesis using RAW cells. However, BMMs used in this study are known to be more sensitive than RAW cells, yielding higher cytotoxicity compared with the results of previous studies, even without statistical differences. In addition, previous studies have reported that BIOD and PMTA have similar properties concerning effects on cell viability.^31^ ^,^ ^32^ This agrees with the findings of this study, which showed BIOD and PMTA had comparable effects on osteoblast cell biocompatibility.

In this study, OCLs derived from BMMs were confirmed to express active TRAP enzyme, a key marker involved in osteoclastic bone resorption. These TRAP positive vesicles may represent a prominent biosynthetic vesicular trafficking route for OCLs to target new synthetic proteins to the ruffled borders.^33^ In OCLs, the enzyme, TRAP, which is specifically expressed, can be used as a marker to detect activated multinucleated OCLs. The results of this study showed TRAP positive multinucleated OCLs were induced at the highest rate in positive controls that matched the previous results.^34^ ^,^ ^35^ Meanwhile, negative controls did not yield TRAP-positive OCLs (Figure 3). Compared with the positive control, CSCs and ALD reduced the accretion of a prominent pool of TRAP positive vesicles; however, there was no significant difference among the three treatments (Figure 3).

OCL differentiation is related to the expression of genes encoding TRAP, CTSK, c-Fos, and other factors.^36^ CTSK is highly expressed in OCLs and plays an important role in bone resorption.^35^ OCLs derived from BMMs exhibit the essential characteristics of OCLs formed *in vivo*, most notably, TRAP and CTSK expression.^37^ The results of mRNA analysis in this study showed PMTA and ALD decreased RANKL/LPS-induced OCL differentiation, consistent with previous studies.^16^ ^,^ ^9^ ^,^ ^19^ ^,^ ^38^ BIOD also inhibited the mRNA expression of osteoclastic markers, but to a lesser extent than PMTA or ALD (P<0.05, Figure 4).

To further delineate the immediate effects of signaling pathways for RANKL/LPS-induced osteoclastogenesis, phosphorylation of IκBα and ERK1/2 was assessed. RANKL interacts with the OCL surface receptor RANK, which recruits tumor necrosis factor receptor-associated factor 6 (TRAF6) to further activate various downstream signaling pathways such as nuclear factor-κB (NF-κB) and three mitogen activated protein kinases (MAPKs) including p38 MAPK, ERK1/2, and c-jun N-terminal kinase.^39^ NF-κB is inactive in the cytosol because it is bound to IκBα, but becomes active once IκBα has been phosphorylated. According to these results, phosphorylation of ERK1/2 was significantly inhibited by CSCs or ALD, suggesting CSCs targeted the MAPK cascade (Figure 6). NF-κB signaling is also downregulated, although phosphorylation of IκBα seemed to be less inhibited compared with ERK1/2. These results suggest downregulation of MAPKs and NF-κB signaling is a mechanism involved in the antiosteoclastogenic activity of CSCs and ALD.

## Conclusions

Within the limitations of this study, we conclude that BIOD and PMTA show an inhibitory effect on OCL differentiation and function similar to alendronate via inhibition of ERK signaling pathways and IκB phosphorylation. The explanation of the precise mechanisms underlying the effect of CSCs and their direct relationship with adjacent tissues over a longer period of time requires further *in vitro* and *in vivo* studies.
